# Inhibitory effect of purple rice husk extract on AFB_1_-induced micronucleus formation in rat liver through modulation of xenobiotic metabolizing enzymes

**DOI:** 10.1186/s12906-019-2647-9

**Published:** 2019-09-03

**Authors:** Arpamas Chariyakornkul, Charatda Punvittayagul, Sirinya Taya, Rawiwan Wongpoomchai

**Affiliations:** 1 Department of Biochemistry, Faculty of Medicine, Chiang Mai University, Chiang Mai, 50200 Thailand; 2 Research Affairs, Faculty of Veterinary Medicine, Chiang Mai University, Chiang Mai, 50100 Thailand; 3 Functional Food Research Center for Well-being, Chiang Mai University, Chiang Mai, 50200 Thailand; 4 Functional Food Research Unit, Science and Technology Research Institute, Chiang Mai University, Chiang Mai, 50200 Thailand

**Keywords:** Aflatoxin B_1_, Liver micronucleus test, Mutagenicity, Rice husk, Xenobiotic metabolizing enzymes

## Abstract

**Background:**

Rice husk, a waste material produced during milling, contains numerous phytochemicals that may be sources of cancer chemopreventive agents. Various biological activities of white and colored rice husk have been reported. However, there are few comparative studies of the cancer chemopreventive effects of white and colored rice husk.

**Methods:**

This study investigated the cancer chemopreventive activities of two different colors of rice husk using in vitro and in vivo models. A bacterial mutation assay using *Salmonella typhimurium* strains TA98 and TA100 was performed; enzyme induction activity in murine hepatoma cells was measured, and a liver micronucleus test was performed in male Wistar rats.

**Results:**

The white rice husk (WRHE) and purple rice husk (PRHE) extracts were not mutagenic in *Salmonella typhimurium* TA98 or TA100 in the presence or absence of metabolic activation. However, the extracts exhibited antimutagenicity against aflatoxin B_1_ (AFB_1_) and 2-amino-3,4 dimethylimidazo[4,5-f]quinolone (MeIQ) in a *Salmonella* mutation assay. The extracts also induced anticarcinogenic enzyme activity in a murine Hepa1c1c7 hepatoma cell line. Interestingly, PRHE but not WRHE exhibited antigenotoxicity in the rat liver micronucleus test. PRHE significantly decreased the number of micronucleated hepatocytes in AFB_1_-initiated rats. PRHE contained higher amounts of phenolic compounds and vitamin E than WRHE in both tocopherols and tocotrienols as well as polyphenol such as cyanidin-3-glucoside, protocatechuic acid and vanillic acid. Furthermore, PRHE increased CYP1A1 and 1A2 activities while decreasing CYP3A2 activity in the livers of AFB_1_-treated rats. PRHE also enhanced various detoxifying enzyme activities, including glutathione *S*-transferase, NAD(P)H quinone oxidoreductase and heme oxygenase.

**Conclusions:**

PRHE showed potent cancer chemopreventive activity in a rat liver micronucleus assay through modulation of phase I and II xenobiotic metabolizing enzymes involved in AFB_1_ metabolism. Vitamin E and phenolic compounds may be candidate antimutagens in purple rice husk.

**Electronic supplementary material:**

The online version of this article (10.1186/s12906-019-2647-9) contains supplementary material, which is available to authorized users.

## Background

Hepatocellular carcinoma (HCC) is the most common cancer worldwide. The most prominent factors associated with HCC include hepatitis B and C viral infection, chronic and heavy alcohol consumption, and fungal toxin contamination. Aflatoxin B_1_ (AFB_1_) is a mycotoxin produced by *Aspergillus* species fungi; the toxin may possibly contaminate human foods. AFB_1_ is the most potent hepatocarcinogen in humans and animals; the toxin is capable of inducing mutations in specific vital genes in hepatocytes, leading to cancer initiation [1]. Xenobiotic metabolizing enzymes (XMEs) in the liver can either activate or detoxify environmental chemicals that are involved in the initiation stage of carcinogenesis [2]. The *Salmonella* mutation assay and micronucleus tests are the standard tests for detecting genotoxic carcinogens [3]. Among the micronucleus tests, the rat liver micronucleus assay is considered as a reliable test for genotoxicants, since the liver is a major source of XMEs [4]. Both bacterial mutation assays and micronucleus tests have been modified for assessing antigenotoxicity of natural products.

The usage of phytochemicals is one of the strategies for decreasing the incidence of various types of cancer. Numerous studies have shown that natural products, both the edible and inedible parts, can act as cancer chemopreventive agents [5]. The secondary metabolites in plants such as phenolic compounds, carotenoids, triterpenoids, alkaloids, and organosulfur compounds are synthesized to protect the plants from hazards in environment; these compounds are also beneficial to animals for preventing diseases. Cancer chemopreventive agents can be divided into two main groups categorized by their mode of action. The first, blocking agents, can inhibit DNA mutation and cancer initiation by modulation of either detoxifying enzymes or the DNA-repairing system. The second, suppressing agents, can delay the development of carcinogenesis by influencing cancer cell proliferation and apoptosis [6].

Rice husk, a waste product from the rice milling process, contains high amounts of phenolic compounds and displays greater biological activity than other parts of rice [7]. Numerous studies have found that rice husk presented antioxidant [7], anti-inflammatory [8] and anti-diabetic activities [9]. White rice husk presented antitumor activity on various cancer cells and inhibited the release of inflammatory cytokines [10, 11]. Since colored rice has become popular due to its beneficial effects on health, the use of colored rice husk waste has also increased. Our previous studies reported that the hydrophilic extracts of purple rice husk extracts presented antimutagenicity against several environmental mutagens in a bacterial model [12]. Moreover, the purple rice husk extracts showed anticlastogenicity against types of hepatocarcinogen-induced micronucleated hepatocyte formation through modulation of detoxifying enzymes [13, 14]. Some phenolic compounds, including anthocyanins, have been proposed to be the anticarcinogens involved; however, the non-phenolic compounds, including gamma-oryzanol and vitamin E, are also suggested as chemopreventive agents. Based on these observations, rice husk is considered as a source of phytochemicals that may exhibit protective activity against carcinogenesis.

At present, there are no reports comparing the chemopreventive properties of white and purple rice husk. Therefore, this study aimed to assess mutagenicity and antimutagenicity of white and purple rice husk extracts using a *Salmonella* mutation assay and a rat liver micronucleus test. The inhibitory mechanism of effective rice husk extract through xenobiotic metabolizing enzyme systems was also evaluated.

## Methods

### Chemicals and reagents

Aflatoxin B_1_ (AFB_1_) and sodium azide (NaN_3_) were obtained from Sigma-Aldrich (St. Louis, USA). 2-Amino-3,4 dimethylimidazo[4,5-f]quinolone (MeIQ), 2-aminoanthracene (2-AA) and 2-(2-furyl)-3-(5-nitro-2-furyl)-acrylamide (AF-2) were purchased from Wako Pure Chemicals (Osaka, Japan). Collagenase type IV and 4′-6-diamidino-2-phenylindole (DAPI) were obtained from Gibco/ Invitrogen Corp. (Carlsbad, USA). The phenolic acid, flavonoid and anthocyanin standards for chemical analysis were high performance liquid chromatography grade. All other chemicals were at least analytical grade.

### Sample extraction

The husks of white rice (San-pah-tawng 1 variety) and purple rice (Kum Doisaket variety) were obtained from rice milling processes at the Mae Hia Agricultural Research Station, Chiang Mai University in August – November 2015. A Genetic stock number (G.S. No.) of San-pah-tawng 1 is 10,479 and deposit at Pathum Thani Rice Research Center, Rice Research and Development Division, Pathum Thani, Thailand. The G.S. Number of Kum Doisaket is under identification. One hundred grams of each rice husk variety were soaked in a liter of absolute methanol at room temperature for 3 days. After filtration using a vacuum pump, the remaining part was re-extracted following the same procedure. Pooled filtrates were concentrated under reduced pressure and vacuum dried to obtain white rice husk extract (WRHE) and purple rice husk extract (PRHE). The extracts were kept at − 20 °C for subsequent experiments.

### Phytochemical contents analysis

Total phenolic compounds and flavonoid content of rice husk extracts were spectrophotometrically determined using the Folin-Ciocalteu technique and aluminium chloride colorimetric method, respectively [14].

The phenolic acids in rice husk extracts were analyzed using reverse-phase HPLC as modified from Chen et al. [15]. The assay conditions were carried out on a reverse-phase C_18_ column (Agilent 4.6 mm × 250 mm, 5 μm) and analyzed using an Agilent HPLC 1260. Gradient elution was done using 3% acetic acid in water and methanol eluents of different compounds. The flow rate and injected volume were 1 ml/min and 10 μl, respectively. The absorbances at 260, 280 and 320 nm were monitored. Phenolic acids contents were defined and calculated using calibration curves of gallic acid, protocatechuic acid, 4-hydroxybenzoic acid, chlorogenic acid, vanillic acid, syringic acid, *p*-coumaric acid, ferulic acid, and ellagic acid. Flavonoid contents were analyzed using reverse-phase HPLC according to Engida et al. with minor modification [16]. The mobile phase consisted of 1% acetic acid in water (A) and 1% acetic acid in methanol (B). Catechin, epicatechin, rutin, quercetin, luteolin, and apigenin were used as the reference standards. The amounts of anthocyanins were analyzed using HPLC conditions as described previously [17]. The amounts of cyanidin-3-glucoside, cyanidin-3-rutinoside, peonidin-3-glucoside and malvidin-3-glucoside were measured using the calibration curves of these external standards.

The γ-oryzanol content in rice husk extracts was examined using a Halo column (0.21 mm × 150 mm, 0.27 μm) and a Hewlett Packard 1100. The mobile phase consisted of 0.5% acetic acid in acetonitrile, methanol and dichloromethane (45:40:15, v/v/v). The flow rate of isocratic elution was 0.1 ml/min, and detection was made at a wavelength of 325 nm [17]. The amount of vitamin E was assayed using a normal phase VertiSep™ UPS silica column (4.6 mm × 250 mm, 5.0 μm), and the mobile phase was composed of hexane, isopropanol, ethyl acetate and acetic acid (97.6: 0.8: 0.8: 0.8, v/v/v/v). The flow rate was 1.0 ml/min, and the analysis was performed at excitation and emission wavelengths of 294 and 326 nm, respectively. The tocopherols (α, β, γ and δ forms) and tocotrienols (α, γ and δ forms) were measured using the calibration curves of external standards [18].

### *Salmonella* mutation assay

Mutagenicity and antimutagenicity tests were performed using *Salmonella typhimurium* TA98 and TA100 in the presence and absence of metabolic activation (±S9) according to Nilnumkhum et al. [13]. The bacterial tester strains were kindly supplied by Dr. Kei-ichi Sugiyama, National Institute of Health, Tokyo, Japan. The 2-AA and AF-2 were used as standard mutagens in the presence and absence of metabolic activation, respectively. The number of revertant colonies was expressed as the mutagenic index (the revertant colonies of the test compound divided by the number of spontaneous revertant colonies). If the mutagenic index was more than 2, the test sample was identified as a possible mutagen.

For the antimutagenicity test, AFB_1_ and MeIQ were used as positive mutagens in strains TA98 and TA100, respectively, in the presence of S9 mix. AF-2 and NaN_3_ were used as positive mutagens in strains TA98 and TA100, respectively, in the absence of S9 mix. The number of revertant colonies was counted by comparing with the specific positive control. The percentage of inhibition was calculated as described previously [19].

### NAD(P)H quinone oxidoreductase (NQO) induction activity in a hepatoma cell line

The NQO-inducing activity was determined in murine hepatoma cells according to Insuan et al. [17]. Briefly, approximately 10,000 cells/well of Hepa1c1c7 cells (ATCC CRL-2026) were seeded onto 96-well plates in alpha minimal essential medium (α-MEM) with 10% fetal bovine serum (FBS) and streptomycin (100 μg/ml), and incubated at 37 °C and 5% CO_2_ for 24 h. The cells were treated with various concentrations of rice husk extracts (0–50 μg/ml) for 24 h. DMSO (0.4%) was used as a negative control, and β-naphthoflavone (0.05 μg/ml) was used as a positive control. Cell density was determined by crystal violet staining, and NQO activity was measured at 620 nm. The concentration required to double the specific activity (CD) value was used as a measure of inducer potency of rice husk extracts.

### Genotoxicity and antigenotoxicity of rice husk extracts in rat liver

Male Wistar rats (50–70 g weight) were purchased from the National Laboratory Animal Center, Mahidol University, Nakhon Pathom, Thailand. Rats were maintained in controlled environments at a temperature of 25 ± 1 °C under a 12 h dark-light cycle and two rats per cage. Water and standard pellet diet were provided ad libitum. The treatment protocol was approved by the Animal Ethics Committee of the Faculty of Medicine, Chiang Mai University (30/2558).

**Fig. 1:**
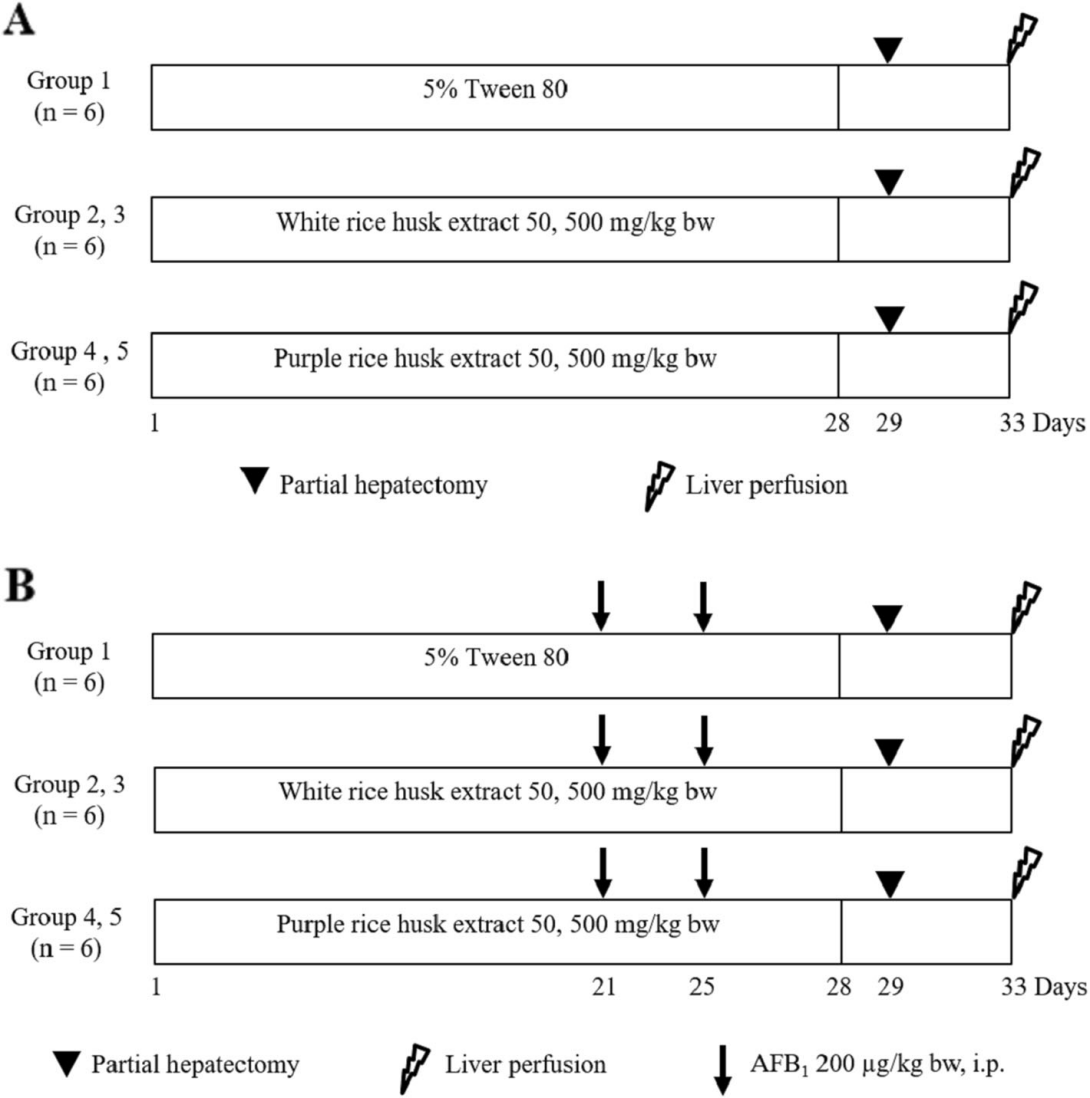
The protocols for (**a**) genotoxicity and (**b**) antigenotoxicity of rice husk extracts in rats

A rat liver micronucleus test was used to determine mutagenicity and antimutagenicity of rice husk extracts in rats. To determine the mutagenic effect of rice husk extracts, male Wistar rats were randomly divided into 5 groups as shown in Fig. 1a. Group 1 received 5% Tween 80 orally as a negative control group. Groups 2 and 3 were fed with WRHE, while groups 4 and 5 were fed with PRHE at concentrations of 50 and 500 mg/kg bw, respectively. These concentrations were 10 and 100 fold lower than of LD_50_ value of PRHE (unpublished data).

Partial hepatectomy was performed to amplify mutated hepatocytes. The derived liver was used for xenobiotic metabolizing enzyme activities analysis. The operation was performed after anesthesia by 4% isoflurane mixed with oxygen inhalation in a closed system until rats were recumbent with loss of righting reflex. Then, anesthesia was rapidly transferred to a nose cone mask to maintain 2% isoflurane in oxygen. Four days after hepatectomy, rats were euthanized by 4% isoflurane mixed with oxygen inhalation in a closed system for at least 5 min at room temperature. Single hepatocytes were isolated via the 2-step collagenase perfusion method [14]. The hepatocytes were stained with DAPI and counted under a fluorescence microscope (× 400), at least 2000 hepatocytes per rat. The scoring criteria of micronucleus hepatocytes were round shape, distinctly stained the same as the main nucleus, and 1/4 lesser diameter than the main nucleus.

To investigate antimutagenicity of rice husk extracts, rats were randomly divided into 5 groups (Fig. 1b). Group 1 was orally fed with 5% Tween 80 as a positive control group. The various doses of WRHE and PRHE were administrated to groups 2–3 and groups 4–5, respectively. All rats were intraperitoneally injected with 200 μg/kg bw of AFB_1_ on days 21 and 25 to induce micronucleated hepatocyte formation. All rats were subjected to partial hepatectomy and liver perfusion. The hepatocytes were stained with DAPI and counted under a fluorescence microscope as described above.

### Preparation of liver cytosolic and microsomal fractions

Rat liver from partial hepatectomy was homogenized in homogenizing buffer and centrifuged at 14,000 rpm for 20 min at 4 °C. The supernatant was then centrifuged at 30,000 rpm for 60 min at 4 °C to obtain a clear supernatant and pellet as cytosolic and microsomal fractions, respectively. The protein concentration of each fraction was examined by the Lowry method using bovine serum albumin (BSA) as a standard.

### Determination of xenobiotic metabolizing enzyme activities in rat liver

The activities of cytochrome P450 (CYP) 1A1, 1A2 and 3A2 were determined by methoxyresorufin-O-demethylation (MROD), ethoxyresorufin-O-deethylation (EROD) and erythromycin N-demethylation (ENDM) methods, respectively, according to Suwannakul, et al. [20]. The activities of CYP1A1 and CYP1A2 were measured with a spectrofluorometer at excitation and emission wavelengths of 520 and 590 nm, respectively, and were expressed as fmol/min/mg protein. The activity of CYP3A2 was measured at a wavelength 405 nm and was expressed as pmol/min/mg protein.

The activity of NADPH-cytochrome P450 reductase (CPR) was investigated according to the rate of cytochrome *c* reduction as described by Punvittayagul et al. [21]. The activity was measured at 550 nm and was calculated using a molar coefficient of 21 mM^− 1^ cm^− 1^. The activity was expressed as units/mg protein.

The glutathione *S-*transferase (GST) activity was analyzed according to Sankam et al. [14]; 1-chloro-2,4-dinitrobenzene was used as a substrate, and the activity was recorded at 340 nm. The activity was calculated by using a molar coefficient of 9.6 M^− 1^ cm^− 1^ and was expressed as units/mg protein.

The UDP-glucuronosyltransferase (UGT) activity was determined according to Summart and Chewonarin with minor modification [22]; *p*-nitrophenol was used as a substrate. The activity was measured at an OD of 405 nm and was expressed as units/mg protein.

The NAD(P)H quinone oxidoreductase (NQO) activity was determined as described previously with minor modification [21]; 2,6 dichlorophenol-indophenol (DCPIP) was used as an electron acceptor. The reduction of DCPIP was measured at an OD of 600 nm and was calculated by using a molar coefficient of 2.1 × 10^4^ M^− 1^ cm^− 1^. The activity was expressed as units/mg protein.

The activity of heme oxygenase (HO) was measured according to Punvittayagul et al. [21]. Hemin was used as a substrate. The enzyme activity was measured at ODs of 460 and 530 nm and was expressed as nmol/min/mg protein.

### Statistical analysis

The results of the *Salmonella* mutation assay were expressed as mean ± SEM. The other data were given as mean ± SD. The significance of differences between groups was determined by one-way ANOVA, and *P* < 0.05 was considered as significant.

## Results

### Phytochemical contents of rice husk extracts

The phytochemical contents of rice husk extracts are shown in Table 1. Purple rice husk extract (PRHE) contained an approximately three fold higher content of total phenolic compounds, including flavonoids, than white rice husk extract (WRHE). The major phenolic acids in PRHE were vanillic acid, *p*-coumaric acid and protocatechuic acid, whereas *p*-coumaric acid and vanillic acid were the main phenolics found in WRHE. Moreover, anthocyanins, including cyanidin-3-glucoside and peonidin-3-glucoside, were only present in the PRHE. In addition, WRHE contained higher amounts of γ–oryzanol, while PRHE contained higher amounts of vitamin E. The major isoform of vitamin E in rice husk extracts was γ–tocotrienol. However, δ–tocotrienol was not detected in either rice husk extract.

**Table 1:** Chemical constituents in methanol extracts of rice husk

Phytochemicals (per gram extract)	White rice husk	Purple rice husk
Spectrophotometric method		
Total phenolic compounds (mg GAE)	37.92 ± 3.35	108.55 ± 14.44*
Total flavonoids (mg CE)	14.76 ± 1.05	46.55 ± 3.27*
HPLC method		
Protocatechuic acid (mg)	ND	3.46 ± 0.00*
Vanillic acid (mg)	1.36 ± 0.20	6.25 ± 0.01*
*p*-Coumaric acid (mg)	3.70 ± 0.04	3.93 ± 0.00*
Epicatechin (mg)	8.25 ± 0.04	6.10 ± 0.03*
Apigenin (mg)	ND	0.82 ± 0.00*
Cyanidin-3-glucoside (mg)	ND	1.96 ± 0.00*
Peonidin-3-glucoside (mg)	ND	1.15 ± 0.01*
Total γ-oryzanol (mg)	4.87 ± 0.13	2.66 ± 0.07*
Total vitamin E (μg)	138.03 ± 2.08	317.99 ± 2.54*
α – tocopherol (μg)	10.69 ± 0.30	61.97 ± 0.21*
β – tocopherol (μg)	27.13 ± 0.15	23.71 ± 0.22*
γ – tocopherol (μg)	19.68 ± 0.29	77.81 ± 0.88*
δ – tocopherol (μg)	ND	24.09 ± 0.12*
α – tocotrienol (μg)	ND	14.55 ± 0.04*
γ – tocotrienol (μg)	80.54 ± 2.21	115.86 ± 2.22*
δ – tocotrienol (μg)	ND	ND

Values expressed as mean ± SD of three independent replicate for spectrophotometric method and independent duplicate for HPLC method. *ND* not detected,

*GAE* gallic acid equivalent, *CE* catechin equivalent

*Significantly different from white rice husk (*p* < 0.05)

### Mutagenicity and antimutagenicity of rice husk extracts in the *Salmonella* mutation assay

WRHE and PRHE did not increase the number of revertant colonies in *S. typhimurium* TA98 or TA100 when compared with the negative control in both the presence and absence of metabolic activation. In addition, various concentrations of rice husk extracts ranging from 40 to 5000 μg/plate did not exhibit cytotoxicity to *S. typhimurium* (Additional file 1: Table S1). The results suggested that WRHE and PRHE were not mutagenic in the bacterial model.

**Fig. 2:**
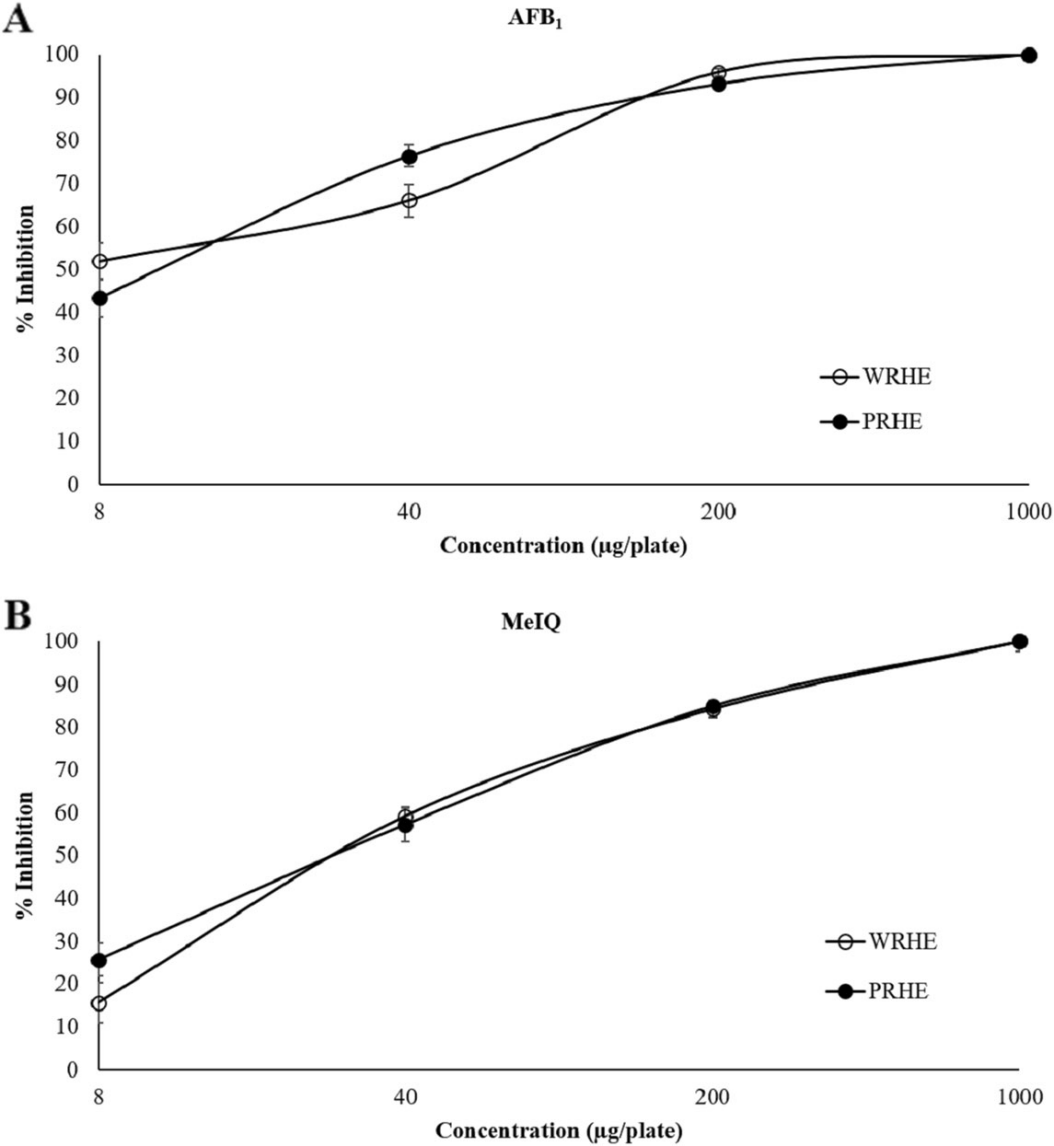
Antimutagenicity of rice husk extracts against (**a**) aflatoxin B_1_ (AFB_1_) and (**b**) 2-amino-3,4 dimethylimidazo[4,5-f]quinoline (MeIQ) using the *Salmonella* mutation assay. Values expressed as mean ± SEM. WRHE: white rice husk extract; PRHE: purple rice husk extract

The highest concentration of rice husk extract used in the antimutagenicity assay was a non-cytotoxic dose, 1000 μg/plate. In the presence of metabolic activation, WRHE and PRHE decreased the number of revertant colonies induced by AFB_1_ in *S. typhimurium* TA 98 and by MeIQ in *S. typhimurium* TA100 in a dose-dependent manner. The percentages of inhibition are shown in Fig. 2. However, rice husk extracts had a weak inhibitory effect on the direct mutagens AF-2 and NaN_3_ in the absence of metabolic activation (Additional file 1: Table S2).

### NQO induction activity of rice husk extracts

**Fig. 3:**
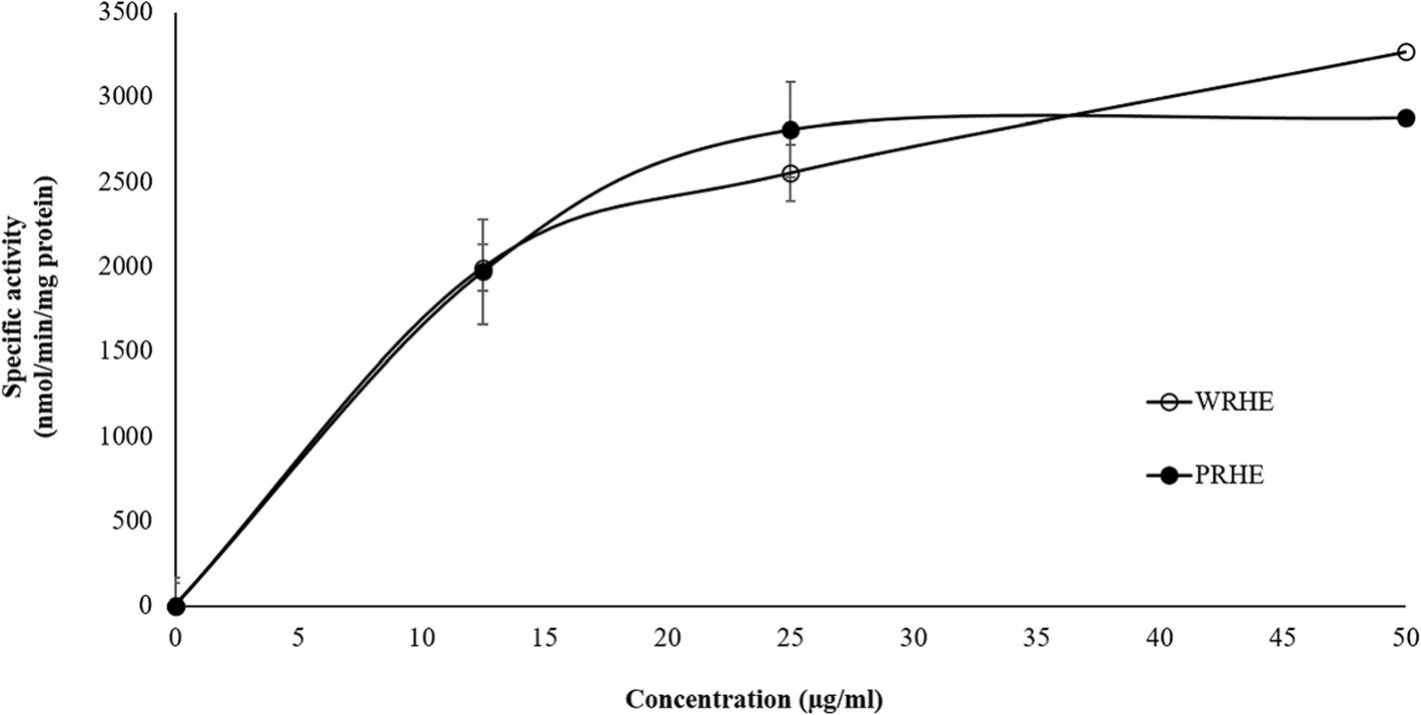
Effect of rice husk extracts on NAD(P)H quinone oxidoreductase inducing activity in the Hepa1c1c7 hepatoma cell line. Values expressed as mean ± SD. WRHE: white rice husk extract; PRHE: purple rice husk extract

Rice husk extracts showed a dose-dependent induction of NQO activity in Hepa1c1c7 cells (Fig. 3). The CD values (the concentration that induces doubling of NQO activity) of WRHE and PRHE were 19.63 ± 1.70 and 18.06 ± 2.41 μg/ml, respectively. The results indicated that rice husk extracts induced anticarcinogenic enzyme activity.

### Genotoxicity and antigenotoxicity of rice husk extracts in rat liver

The genotoxic and antigenotoxic effects of rice husk extracts are summarized in Table 2. The treatments of 50 and 500 mg/kg bw of WRHE and PRHE for 28 days did not increase the incidence of micronucleated hepatocytes, binucleated hepatocytes or the mitotic index when compared with the control group. These results demonstrated that rice husk extract was not genotoxic to rats.

**Table 2:** Genotoxicity and antigenotoxicity of rice husk extracts in rat liver

Treatments	Number per 1000 hepatocytes		Mitotic index	% Inhibition
	MNH	BNH cells		
5% Tween-80	2.6 ± 0.8	2.4 ± 0.4	1.0 ± 0.2	–
WRHE 50 mg/kg bw	2.8 ± 1.3	2.3 ± 0.5	1.0 ± 0.4	**–**
WRHE 500 mg/kg bw	2.8 ± 0.9	2.1 ± 0.4	0.7 ± 0.2	–
PRHE 50 mg/kg bw	3.3 ± 0.2	1.9 ± 0.2	0.8 ± 0.3	**–**
PRHE 500 mg/kg bw	3.0 ± 0.9	2.2 ± 0.3	0.9 ± 0.2	**–**
AFB_1_ + 5% Tween-80	9.7 ± 2.7*	4.4 ± 0.5*	3.0 ± 0.6*	–
AFB_1_ + WRHE 50 mg/kg bw	7.2 ± 1.4	3.6 ± 0.1	2.4 ± 0.3	26.0
AFB_1_ + WRHE 500 mg/kg bw	6.8 ± 0.9	4.3 ± 0.6	2.6 ± 0.5	30.0
AFB_1_ + PRHE 50 mg/kg bw	5.6 ± 1.9^#^	4.1 ± 0.3	2.4 ± 0.1	42.3
AFB_1_ + PRHE 500 mg/kg bw	5.4 ± 1.8^#^	4.0 ± 0.5	2.3 ± 0.3	44.7

Values expressed as mean ± SD, *n* = 6

*Significant difference from 5% Tween-80 (*p* < 0.05), ^#^Significant difference from AFB_1_ + 5% Tween 80 (*p* < 0.05)

*MNH* micronucleated hepatocytes, *BNH* binucleated hepatocytes, *AFB*_1_ Aflatoxin B_1_,

*WRHE* white rice husk extract, *PRHE* purple rice husk extract

We evaluated the antigenotoxic effects of rice husk extracts against AFB_1_-induced micronucleus formation in rat liver. AFB_1_ significantly increased the number of micronucleated hepatocytes, binucleated hepatocytes and mitotic cells compared to the negative control group. Interestingly, oral administration of 50 and 500 mg/kg bw of PRHE significantly diminished the number of micronucleated hepatocytes in AFB_1_-initiated rats with inhibition of 42.3 and 44.7%, respectively. WRHE slightly reduced the number of micronucleated hepatocytes induced by AFB_1_ but showed no significant difference when compared with the AFB_1_ treated group. These results suggested that PRHE was more efficient than WRHE in inhibiting genotoxicity induced by AFB_1_.

### Effect of rice husk extracts on the activity of xenobiotic metabolizing enzymes in rat liver

Table 3 shows that the low dose (50 mg/kg bw) of PRHE significantly decreased the activity of CYP3A2, while the low dose of WRHE did not affect either phase I or II enzymes. In addition, the high dose (500 mg/kg bw) of WRHE significantly decreased the activity of CYP3A2, whereas the high dose of PRHE significantly enhanced CYP1A1 activity and decreased the activity of NQO. Neither WRHE nor PRHE influenced the activities of CYP1A2, CPR, GST, UGT or HO.

**Table 3:** Effect of rice husk extracts on xenobiotic metabolizing enzyme activities in the liver of rats

Enzyme activities (per mg protein)	5% Tween-80	White rice husk		Purple rice husk	
		50 mg/kg bw	500 mg/kg bw	50 mg/kg bw	500 mg/kg bw
CYP1A1 (fmol/min)	15.48 ± 6.66	9.49 ± 3.93	12.79 ± 6.50	9.86 ± 1.57	23.69 ± 5.20*
CYP1A2 (fmol/min)	13.80 ± 4.45	10.49 ± 5.53	14.60 ± 8.51	12.65 ± 6.15	16.22 ± 4.41
CYP3A2 (pmol/min)	16.48 ± 6.42	12.77 ± 1.58	9.99 ± 2.81*	9.99 ± 3.22*	12.60 ± 2.54
Cytochrome P450 reductase (× 10^− 3^ Unit)	17.13 ± 3.90	24.93 ± 5.99	25.26 ± 9.98	22.15 ± 9.00	17.62 ± 8.90
Glutathione *S*-transferase (× 10^− 2^ Unit)	41.70 ± 8.67	40.82 ± 7.62	47.81 ± 8.81	43.22 ± 4.03	44.49 ± 4.60
UDP-glucuronosyltransferase (× 10^− 3^ Unit)	31.13 ± 2.79	31.04 ± 3.84	36.82 ± 10.36	26.63 ± 4.54	34.60 ± 7.32
NAD(P)H-quinone oxidoreductase (× 10^− 3^ Unit)	2.68 ± 0.62	2.04 ± 0.47	2.26 ± 0.95	2.21 ± 0.35	1.82 ± 0.46*
Heme oxygenase (nmol/min)	5.10 ± 0.59	4.18 ± 1.56	4.38 ± 1.14	3.93 ± 0.52	5.61 ± 1.28

Values expressed as mean ± SD, *n* = 6, *Significant difference from 5% Tween-80 (*p* < 0.05)

**Fig. 4:**
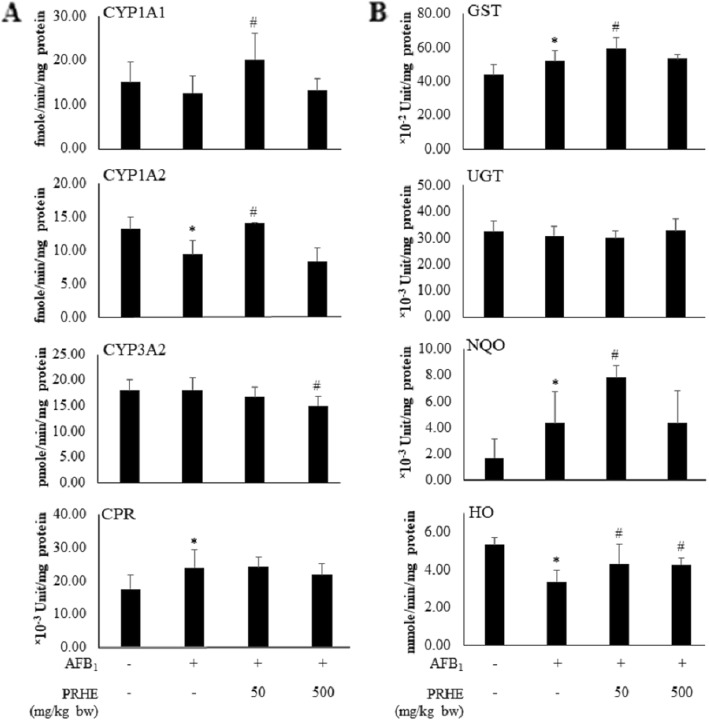
Effect of purple rice husk extract on activities of xenobiotic metabolizing enzymes in the liver of AFB_1_-initiated rats. (**a**) phase I xenobiotic metabolizing enzymes, (**b**) phase II xenobiotic metabolizing enzymes. Values expressed as mean ± SD, *n* = 6. AFB_1_: aflatoxin B_1_; PRHE: purple rice husk extract; CYP: cytochrome P450; CPR: cytochrome P450 reductase; GST: glutathione *S*-transferase; UGT: UDP-glucuronyltransferase; NQO: NAD(P)H quinone oxidoreductase; HO: heme oxygenase. * Significant difference from control group (*p* < 0.05). ^#^ Significant difference from AFB_1_-treated group (*p* < 0.05)

PRHE at doses of 50 and 500 mg/kg bw inhibited micronucleated hepatocyte formation initiated by AFB_1_. The treatment with AFB_1_ alone significantly reduced the activities of CYP1A2 and HO but induced CPR, GST and NQO activities compared with the negative control. The low dose of PRHE significantly increased the activities of CYP1A1, CYP1A2, GST, NQO and HO compared with the AFB_1_ alone group. Moreover, high dose of PRHE significantly decreased CYP3A2 and increased HO activities in rat liver. However, neither AFB_1_ alone nor AFB_1_ combined with PRHE affected the activity of the UGT enzyme. The results are summarized in Fig. 4.

## Discussion

Prevention of DNA mutation is one of the chemopreventive approaches to reducing cancer incidence [6]. Not only anthocyanins but also some non-anthocyanin phenolic compounds and non-phenolic compounds have been identified as cancer chemopreventive agents. The *Salmonella* mutation assay and NQO induction assay were used as cancer chemoprevention screening methods of rice husk extracts. Results showed that both WRHE and PRHE suppressed AFB_1_- and MeIQ-induced mutagenesis in *Salmonella*. These mutagens need CYP450 to express their genotoxicity. The extracts also enhanced the activity of an anticarcinogenic enzyme, NAD(P)H-quinone oxidoreductase, in a murine hepatoma cell line. There was no significant difference between WRHE and PRHE in both in vitro assays. Therefore, we further determined the antimutagenicity of both rice husk extracts against AFB_1_ treated rats. PRHE (but not WRHE) exhibited antimutagenicity in the livers of AFB_1_-treated rats. This may indicate that the antigenotoxicity of the rice husk extracts depended on xenobiotic metabolism.

Phytochemicals are secondary metabolites such as phenolic acids, flavonoids, alkaloids and terpenoids that are produced by plants and that exhibit various biological and pharmacological activities [5]. In this study, the cancer chemopreventive activity of PRHE was stronger than that of WRHE. PRHE not only contained anthocyanins that gave the purple husk its dark color but also contained higher amounts of vitamin E and phenolic compounds. Several studies reported that tocopherols and tocotrienols could inhibit colon, prostate, mammary and lung tumorigenesis in animal models [23–25]. Phenolic compounds including anthocyanins have also been shown to possess antioxidant, antimicrobial, anti-inflammatory and anticancer activities [26, 27]. Our previous study found that vanillic acid, which is a predominant phenolic acid in purple rice husk, presented antimutagenicity against AFB_1_-initiated rat hepatocarcinogenesis [13]. Vanillic acid has also exhibited anticancer activities against several cancer cell lines [28]. Moreover, some anthocyanins, including cyanidin-3-glucoside, decreased tumor numbers in azoxymethane-induced colon cancer [29]. This study also showed that protocatechuic acid, a major metabolite of anthocyanins, was present in colored rice husk but not in white rice husk. Protocatechuic acid inhibited cancer cell growth and exerted pro-apoptotic and anti-proliferative effects in different tissues [30]. Although γ-oryzanol exhibited cancer chemopreventive activity [23], the level found in WRHE, which was higher than in PRHE in this study, might not reach the antimutagenic dose for inhibiting micronucleus formation in the initiation stage of AFB_1_-induced hepatocarcinogenesis. Vitamin E was presumably one of lipophilic chemopreventive agents present in purple rice husk, while cyanidin and peonidin glucosides, protocatechuic acid and vanillic acid were the candidate hydrophilic antimutagens in purple rice husk.

AFB_1_, the most mutagenic and carcinogenic form of aflatoxin, is principally metabolized by CYP1A2 and 3A2 in the rat liver to form AFB_1_–8,9-epoxide. The epoxide can bind with guanine in DNA, resulting in AFB_1_-N^7^-guanine and AFB_1_-formamidopyrimidine. These adducts provoke DNA mutations, particularly in codons 12 and 13 of ras oncogenes, leading to hepatocellular carcinoma formation in rats [31]. AFB_1_ is also metabolized by several CYP families to hydroxylated metabolites such as AFM_1_ and AFQ_1_ that are less toxic. In this study, we found that the patterns of several phase I and II metabolizing enzyme activities differed from those observed in other studies of AFB_1_ metabolism [32, 33]. This may have been due to differences in the timing of AFB_1_ administration.

PRHE significantly decreased micronucleated hepatocyte formation initiated by AFB_1_ in rats. GST plays a major role in the detoxification pathway of AFB_1_, and we found that PRHE induced the activity of GST and other detoxifying enzymes, including NQO and HO. These effects could prevent the ultimate AFB_1_ accumulation and reduce either DNA or protein adduct formation. GST, NQO and HO are regulated by NF-E2 related factor 2 (Nrf-2), a transcription factor that is important in the maintenance of cellular antioxidant responses and xenobiotic metabolism [34]. It has been suggested that some phytochemicals in PRHE may up-regulate Nrf-2 expression, resulting in induction of detoxifying and antioxidant enzymes that contribute to AFB_1_ detoxification. Several studies have shown that phenolic acids, flavonoids and anthocyanins can activate the cellular antioxidant system via the Nrf-2 signaling pathway [35].

Miao et al. reported that the transcription of Nrf2-regulated genes is directly modulated by aryl hydrocarbon receptor (AhR), which regulates transcription of CYP1A families [36]. This interaction represents a cross-talk between AhR and Nrf2 pathways, thereby contributing to more effective phase I and II enzyme activities. It is possible that PRHE affected these two pathways, resulting in increased activity of CYP1As and phase II enzymes. PRHE may protect against AFB_1_-induced mutagenesis in the rat liver through enhancement of the CYP1A family, which would accelerate production of epoxide and hydroxylated metabolites as the substrates for the further phase and induction of detoxifying and antioxidant enzymes to eliminate polar AFB_1_ metabolites. Nevertheless, the antimutagenicity of PRHE against AFB_1_ in the rat liver was not dose dependent, and the responses to xenobiotic metabolizing enzymes varied. Furthermore, both rice husk extracts scarcely altered hepatic metabolizing enzymes of rats in physiological conditions. It is possible that the phytochemicals in PRHE might present hormetic responses, with low doses protecting against cellular stress by induction of Nrf-2 and AhR downstream target genes, while high doses may contribute to triggering of initiated cell death [37].

## Conclusions

Purple rice husk extract exhibited potent cancer chemopreventive properties using in vitro and in vivo assessment. It ameliorated AFB_1_-induced micronucleus formation in rat liver via modulation of some xenobiotic metabolizing enzymes involving in AFB_1_ metabolism. Vitamin E and phenolic compounds including anthocyanins might act as antimutagens in purple rice husk.

## Additional file

Additional file 1: **Table S1.** Mutagenicity of rice husk extracts in *Salmonella typhimurium* strains TA98 and TA100 in the absence and presence of metabolic activation. Values expressed as mean ± SEM. 2AA: 2-aminoanthracene; AF2: 2-(2-furyl)-3-(5-nitro-2-furyl)acrylamide; WRHE: white rice husk extract; PRHE: purple rice husk extract. **Table S2.** Antimutagenicity of rice husk extracts in *Salmonella typhimurium* strains TA98 and TA100 in the absence of metabolic activation. Values expressed as mean ± SEM. AF2: 2-(2-furyl)-3-(5-nitro-2-furyl)acrylamide; NaN_3_: sodium azide; WRHE: white rice husk extract; PRHE: purple rice husk extract. (DOCX 20 kb)

## Data Availability

All data generated or analyzed during this study are included in this published article.

## Abbreviations

2-AA: 2-aminoanthracene
AFB_1_: Aflatoxin B_1_
AhR: Aryl hydrocarbon receptor
BNH: Binucleated hepatocytes
BSA: Bovine serum albumin
CPR: NADPH-cytochrome P450 reductase
CYP: Cytochrome P450
DAPI: 4′,6-diamidino-2-phenylindole
DCPIP: 2,6-dichlorophenol-indolephenol
ENDM: Erythromycin-N-demethylation
EROD: Ethoxyresorufin-O-deethylation
FBS: Fetal bovine serum
GST: Glutathione *S-*transferase
HCC: Hepatocellular carcinoma
HO: Heme oxygenase
HPLC: High performance liquid chromatography
MNH: Micronucleated hepatocytes
MROD: Methoxyresorufin-O-demethylation
NQO: NAD(P)H quinone oxidoreduxtase
Nrf-2: NF-E2 related factor 2
PH: Partial hepatectomy
PRHE: Puple rice husk extract
UGT: UDP-glucuronosyltransferase
WRHE: White rice husk extract
XMEs: Xenobiotic metabolizing enzymes
α – MEM: alpha minimal essential medium
