# Supplementary material for: Inhibitory effect of purple rice husk extract on AFB1-induced micronucleus formation in rat liver through modulation of xenobiotic metabolizing enzymes
Source: BMC Complement Altern Med. 2019 Sep 3;19:237. doi: 10.1186/s12906-019-2647-9 (PMC6724366; doi:10.1186/s12906-019-2647-9)
Supplement: Supplementary file 1 — Table S1. Mutagenicity of rice husk extracts in Salmonella typhimurium strains TA98 and TA100 in the absence and presence of metabolic activation. Values expressed as mean ± SEM. 2AA: 2-aminoanthracene; AF2: 2-(2-furyl)-3-(5-nitro-2-furyl)acrylamide; WRHE: white rice husk extract; PRHE: purple rice husk extract. Table S2. Antimutagenicity of rice husk extracts in Salmonella typhimurium strains TA98 and TA100 in the absence of metabolic activation. Values expressed as mean ± SEM. AF2: 2-(2-furyl)-3-(5-nitro-2-furyl)acrylamide; NaN3: sodium azide; WRHE: white rice husk extract; PRHE: purple rice husk extract. (DOCX 20 kb) [file 12906_2019_2647_MOESM1_ESM.docx]

**Additional file 1**

**Table S1.** Mutagenicity of rice husk extracts in *Salmonella typhimurium* strains TA98 and TA100 in the absence and presence of metabolic activation.

| **Samples** | **Concentration** | **His^+^ revertant colonies per plate (Mutagenic index: MI)** | | | |
| --- | --- | --- | --- | --- | --- |
|  |  | **TA 98** | | **TA 100** | |
|  |  | **+ S9** | **- S9** | **+ S9** | **- S9** |
| DMSO | - | 33 ± 2.0 (1.0) | 35 ± 2.1 (1.0) | 123 ± 4.0 (1.0) | 134 ± 4.8 (1.0) |
| 2AA | 0.5 µg/pl | 986 ± 32.8 (30.5) | - | 808 ± 31.4 (6.6) | - |
| AF2 | 0.1 µg/pl | - | 397 ± 17.4 (11.4) | - | - |
| AF2 | 0.01 µg/pl | - | - | - | 689 ± 31.8 (5.1) |
| WRHE | 0.04 mg/pl | 38 ± 2.2 (1.2) | 31 ± 1.4 (0.9) | 114 ± 6.0 (0.9) | 136 ± 0.8 (1.0) |
|  | 0.2 mg/pl | 37 ± 2.5 (1.1) | 32 ± 1.8 (0.9) | 101 ± 2.2 (0.8) | 122 ± 7.7 (0.9) |
|  | 1 mg/pl | 34 ± 1.6 (1.1) | 33 ± 1.6 (1.0) | 100 ± 2.7 (0.8) | 104 ± 4.3 (0.8) |
|  | 5 mg/pl | 35 ± 1.5 (1.1) | 31 ± 2.2 (0.9) | 115 ± 10.7 (1.0) | 95 ± 2.4 (0.7) |
| PRHE | 0.04 mg/pl | 34 ± 2.3 (1.1) | 35 ± 1.6 (1.0) | 115 ± 2.7 (0.9) | 128 ± 4.5 (1.0) |
|  | 0.2 mg/pl | 36 ± 1.3 (1.1) | 28 ± 1.6 (0.8) | 113 ± 2.7 (0.9) | 123 ± 4.6 (0.9) |
|  | 1 mg/pl | 32 ± 2.9 (1.0) | 32 ± 2.5 (0.9) | 109 ± 4.6 (0.9) | 110 ± 4.9 (0.8) |
|  | 5 mg/pl | 34 ± 2.1 (1.1) | 36 ± 2.2 (1.0) | 115 ± 2.4 (0.9) | 93 ± 1.8 (0.7) |

Values expressed as mean ± SEM.

2AA: 2-aminoanthracene; AF2: 2-(2-furyl)-3-(5-nitro-2-furyl)acrylamide; WRHE: white rice husk extract; PRHE: purple rice husk extract.

**Table S2.** Antimutagenicity of rice husk extracts in *Salmonella typhimurium* strains TA98 and TA100 in the absence of metabolic activation.

| **Samples** | **Concentration** | **His^+^ revertant colonies per plate**  **(% inhibition)** | |
| --- | --- | --- | --- |
|  |  | **TA98** | **TA100** |
| DMSO | - | 26 ± 0.7 | 121 ± 5.0 |
| AF2 | 0.1 µg/pl | 286 ± 8.9 | - |
| NaN_3_ | 1 ug/pl | - | 485 ± 10.0 |
| WRHE | 0.008 mg/pl | 264 ± 11.8 (8.5) | 433 ± 5.6 (12.6) |
|  | 0.04 mg/pl | 279 ± 14.5 (3.1) | 426 ± 6.9 (14.7) |
|  | 0.2 mg/pl | 277 ± 10.1 (3.3) | 397 ± 7.8 (22.7) |
|  | 1 mg/pl | 222 ± 8.1 (24.4) | 329 ± 10.6 (41.7) |
| PRHE | 0.008 mg/pl | 271 ± 9.4 (5.6) | 444 ± 6.7 (9.6) |
|  | 0.04 mg/pl | 288 ± 10.7 (-1.1) | 443 ± 6.0 (9.7) |
|  | 0.2 mg/pl | 291 ± 8.5 (-2.0) | 402 ± 14.1 (21.5) |
|  | 1 mg/pl | 262 ± 8.4 (8.9) | 352 ± 15.4 (35.4) |

Values expressed as mean ± SEM.

AF2: 2-(2-furyl)-3-(5-nitro-2-furyl)acrylamide; NaN_3_: sodium azide; WRHE: white rice husk extract; PRHE: purple rice husk extract.
